# When Should “Pre” Carry as Much Weight in the Diabetes Comorbidity Debate? Insights From a Population-Based Survey

**DOI:** 10.5888/pcd15.170158

**Published:** 2018-03-22

**Authors:** Negin Iranfar, Tyler C. Smith

**Affiliations:** 1 National University, San Diego, California

## Abstract

**Introduction:**

Estimates indicate that 86 million people in the United States fit the clinical definition of prediabetes, which contributes to the epidemic of nearly 2 million new diagnoses of type 2 diabetes mellitus each year. Effort has focused on preventing prediabetes from progressing to clinical diabetes. We investigated the sociodemographic, behavioral, and health factors in people diagnosed with diabetes or prediabetes and associated leading indicators and comorbidities.

**Methods:**

We used Behavioral Risk Factor Surveillance System data from 2011 through 2015 (N = 1,699,754). All respondents aged 18 years or older with complete covariate data were included, differentiating between self-reported diagnosis of diabetes or prediabetes. Weighted univariate and multivariable logistic regression analyses of 28 variables were developed, with adjusted odds of diagnosis, and standardized coefficients were calculated to rank predictors for diabetes and prediabetes.

**Results:**

Prevalence of prediabetes increased each year between 2011 and 2014. After adjusting for demographic, lifestyle, and health variables, the most significant predictors in magnitude of importance for prediabetes and diabetes were age and body mass index. Although adjusted odds for cardiovascular disease and kidney disease were higher in respondents with diabetes than in those with prediabetes, respondents with prediabetes had higher adjusted odds of arthritis, depressive disorder, cancer, and chronic obstructive pulmonary disease.

**Conclusions:**

Concurrent chronic diseases occur in people with prediabetes even at normal and overweight classifications. By identifying the conditions that are concomitant with diabetes, people with prediabetes can be provided with more rigorous and individualized treatments that can lead to better population health.

## Introduction

Type 2 diabetes mellitus is a multifactorial chronic condition caused by defects in the metabolic system relating to insulin secretion and insulin resistance (1). According to the Centers for Disease Control and Prevention (CDC), an estimated 1.7 million incident cases of diabetes among Americans aged 20 years or older were reported in 2012, an equivalent of 4,657 daily cases (2). In 2014, the World Health Organization estimated that 387 million people worldwide have type 2 diabetes, with half as many cases still undiagnosed, projecting prevalence of type 2 diabetes at 592 million by 2035 (3).

The global epidemic of type 2 diabetes was predicted as early as 1971, as the result of a rapid increase in the prevalence of this disease among indigenous populations who adopted Western lifestyles (4,5). Over the past 4 decades, many epidemiological studies demonstrated that the Western way of life has contributed to the increased prevalence of type 2 diabetes and its complications (6–8). Prediabetes is an early stage of dysglycemia that occurs before diagnosis of overt diabetes (9). According to CDC, as of 2014, one in 3 adults older than 20 years (86 million people) had clinical prediabetes, with an estimated 8% to 12% diagnosed (2,^10^); without any intervention to treat prediabetes through lifestyle modification, medication, or both, 5% to 10% of them will progress to type 2 diabetes each year, compared with 2% of normoglycemic people (2,^9^,^11^). We used data from a large, representative, cross-sectional national survey to investigate the trend in type 2 diabetes and prediabetes from 2011 through 2015 in the United States.

## Methods

### Population and data source

Our study used a serial cross-sectional design using Behavioral Risk Factor Surveillance System (BRFSS) survey data from 2011 through 2015, for adults aged 18 or older with complete covariate data. BRFSS is an annual survey of randomly selected US residents contacted via telephone landline and cellular telephone in all 50 states, the District of Columbia, and 3 US territories, collected in either English or Spanish (12). Only 1 member of each household is surveyed, and the data are valid, reliable, and generalizable to the US population (13). The average response rate for the 2011 through 2015 BRFSS was 48%. BRFSS data are publicly available and contain no personal identifiers; for this reason, this study was determined to be exempt from review by the National University Institutional Review Board.

### Variables

#### Measures

We used data for respondents who self-reported a diagnosis of diabetes or prediabetes for whom there were full covariant data, based on their answers to 2 questions: 1) “Have you ever been told by a doctor or a health provider you have diabetes?”; and 2) “Ever been told by a doctor or a health provider you have prediabetes or borderline diabetes?” The aggregate 5-year affirmative responses for the questions were 1) n = 215,441 (12.7%; weighted frequency 10.5%) and 2) n = 63,567 (3.7%; weighted frequency 3%). Women who self-reported having diabetes or prediabetes during pregnancy (gestational diabetes) were excluded from this study. Clinically, prediabetes is defined as the condition where glycemic parameters are above normal but below diabetes thresholds (14). The American Diabetes Association describes prediabetes as fasting plasma glucose (FPG) of 5.6 to 6.9 mmol/L, referred to as an impaired fasting glucose level, and/or postload plasma glucose level of 7.8 to 11.1 mmol/L, referred to as impaired glucose tolerance (14), or hemoglobin A_1c_ levels of 5.7% to 6.4% (15). We used data on respondents who self-reported a diagnosis of type 2 diabetes or prediabetes.

#### Demographic and socioeconomic factors

Self-reported age in years (18–34, 35–49, 50–64, or ≥65), marital status (married, never married, or other), military veteran status (yes or no), education level (≤high school graduate, or some college and above), annual household income (<$15,000, $15,000 to <$25,000, $25,000 to <$35,000, $35,000 to <$50,000, or ≥$50,000), consistent access to health provider (yes or no), routine annual checkup in the past 12 months (yes or no), race/ethnicity (white non-Hispanic, black non-Hispanic, Hispanic, or other), and sex. Data were also analyzed by geographic regions according to the 9 US Census Bureau designations (16): Northeast (New England division and Middle Atlantic division); Midwest (East North Central division and West North Central division); South (South Atlantic division, East South Central division, and West South Central division); and West (Mountain division and Pacific division) (for detailed list of states included in each division see https://www2.census.gov/geo/pdfs/maps-data/maps/reference/us_regdiv.pdf).

#### Health variables

Self-reported body mass index (BMI) was stratified into 4 categories: underweight (BMI <18.5 kg/m^2^ [weight in kg divided by height in m^2^]), normal weight (BMI, 18.5–24.9), overweight (BMI 25.0–30.0), or obese (BMI >30.0). Other variables were general health condition (excellent/very good, good/fair, or poor); limited activity because of physical, mental, or emotional health (yes or no); and ever diagnosed with any of the following health conditions (yes or no): arthritis (eg, rheumatoid arthritis, gout, lupus, fibromyalgia), depressive disorder, asthma, chronic obstructive pulmonary disease (COPD) or pulmonary disease, kidney disease, cancer, or cardiovascular disease (CVD) (including chronic heart disease, heart attack, and stroke).

#### Lifestyle variables

Binary questions (yes or no) included smoking (100 or more cigarettes in lifetime), habitual drinking (men >14 drinks/week, women >7 drinks/week), and habitual exercise (any physical activity or exercise other than daily work-related routine in the past 30 days).

### Statistical analysis

Descriptive and univariate analyses of the study population, prediabetes, and diabetes were conducted for all variables (*P* < .05 to assess significance). BRFSS weighting was used to adjust for differences in noncoverage and nonresponse in the sample to produce more generalizable estimates (17). Weighted multivariable logistic regression controlling for demographic, health, and lifestyle variables was used to obtain weighted and adjusted odds ratios (AORs) and 95% confidence intervals (CIs) for each variable with respect to prediabetes and diabetes. A multicollinearity assessment, using a variance inflation factor, was performed, with values 4 and above indicating collinearity. Fisher scoring algorithm was used to calculate maximum likelihood and identify the most influential factors in diabetes and prediabetes. Statistical analysis and data management were performed by using SAS software, version 9.4 (SAS Institute, Inc.).

## Results

All year-to-year differences in frequency distribution for each variable for the study population (N = 1,699,754) were significant, except for sex (*P* = .19) (Table 1). Most health conditions included in this study did not show a substantial increase in prevalence in the 5-year period, with 2 exceptions: depressive disorder increased from 16.9% to 18.1%, and obesity increased from 28.4% to 29.8%. A reduction in smoking occurred, from 45.4% in 2011 to 42.5% in 2015. The prevalence of education beyond high school increased from 58.7% to 61.3%; households with annual incomes from $15,000 to less than $25,000 decreased slightly from 17.6% to 15.8%; households with annual incomes greater than $50,000 increased from 44.9% to 49.9% (all values 2011, 2015, respectively). During 2011 through 2015, regular annual physical checkups increased from 67.0% to 70.0%; more than half of the population steadily reported a self-perceived health condition as excellent or very good (5-year average 52.6%); 42.7% reported good to fair, and 4.6% reported poor general health (*P* = .03) (Table 1).

**Table 1:** Characteristics of Respondents (N = 1,699,754) Who Responded Yes for Condition or Behavior, Behavioral Risk Factor Surveillance System, 2011–2015^a^

Characteristic	2011, %	2012, %	2013, %	2014, %	2015, %	*P* Value^b^
**Health Condition**						
**Chronic condition**						
Arthritis^c^	25.4	26.2	26.1	26.6	25.7	<.001
Depressive disorder	16.9	17.1	18.0	18.3	18.1	<.001
Asthma	13.3	13.0	13.8	13.5	13.6	<.001
Cancer^d^	11.6	11.3	11.8	11.6	12.1	<.001
Cardiovascular disease^e^	8.4	8.7	8.8	8.9	8.6	<.001
Chronic obstructive pulmonary disease (COPD)^f^	6.4	6.5	6.6	6.8	6.6	.001
Kidney disease	2.5	2.7	2.7	2.8	2.7	.01
Limited activity^g^	23.8	20.5	20.0	21.0	20.7	<.001
**General health**						
Excellent, very good	52.6	52.6	52.5	52.8	52.8	.03
Good, fair	42.8	42.6	42.9	42.5	42.8	
Poor	4.6	4.8	4.6	4.7	4.4	
**BMI^h^**						
Underweight	1.6	1.7	1.7	1.8	1.5	<.001
Normal	33.9	33.7	33.2	32.8	32.5	
Overweight	36.1	36.1	35.9	35.6	36.2	
Obese	28.4	28.6	29.2	29.8	29.8	
**Lifestyle**						
**Smoking^i^**	45.4	44.2	43.5	42.8	42.5	<.001
**Consume alcohol regularly^j^**	6.8	6.2	6.3	6.2	6.2	<.001
**Exercise regularly^k^**	75.4	77.8	74.7	77.5	75.2	<.001
**Demographics/socioeconomics**						
**Military status**						
Veteran	12.2	12.0	11.8	12.5	12.2	<.001
Nonveteran	87.8	88.0	88.2	87.5	87.8	
**Education level**						
High school graduate or less	41.3	40.8	40.2	40.1	38.7	<.001
Some college and above	58.7	59.2	59.8	59.9	61.3	
**Marital status**						
Married	55.3	54.1	56.1	55.3	55.4	<.001
Never married	24.0	25.2	22.7	23.6	23.2	
Other^l^	20.7	20.7	21.2	21.2	21.3	
**Sex**						
Male	50.6	50.5	50.3	50.4	50.9	.19
Female	49.4	49.5	49.7	49.6	49.1	
**Race/ethnicity**						
White non-Hispanic	69.7	67.5	67.6	68.1	67.5	<.001
Black non-Hispanic	11.2	11.8	11.5	11.8	11.4	
Hispanic	12.1	13.2	13.5	12.7	13.5	
Other	7.0	7.5	7.3	7.3	7.6	
**Income, $**						
<15,000	11.9	12.2	11.9	11.4	10.2	<.001
15,000 to <25,000	17.6	17.3	17.0	16.9	15.8	
25,000 to <35,000	11.4	11.0	10.8	10.7	10.3	
35,000 to <50,000	14.3	14.4	14.3	13.9	13.8	
≥50,000	44.9	45.1	46.0	47.1	49.9	
**Health care access**						
Consistent access to health provider	80.1	79.4	78.1	79.0	80.4	<.001
Annual health checkup	67.0	67.6	68.5	70.1	70.0	<.001
**Age, y**						
18–34	27.0	27.4	26.9	27.3	26.8	<.001
35–49	27.9	26.6	25.7	25.2	25.1	
50–64	27.5	27.9	28.6	28.3	28.2	
≥65	17.5	18.1	18.8	19.2	19.8	
**Geographic region^m^**						
Midwest: East North Central division	16.2	15.9	16.0	16.2	16.0	<.001
Midwest: West North Central division	6.9	6.8	6.4	6.9	6.8	
South: South Atlantic division	19.6	19.7	19.7	20.0	19.9	
South: East South Central division	5.7	5.9	5.5	6.0	5.8	
South: West South Central division	11.3	11.5	11.1	11.2	11.3	
Northeast: New England division	4.8	4.7	4.7	4.7	4.4	
Northeast: Middle Atlantic division	12.9	12.7	12.9	13.2	12.8	
West: Mountain division	7.1	7.0	7.2	7.2	7.1	
West: Pacific division	15.5	15.8	16.4	14.6	15.9	

a Percentages are weighted.

b *P* values based on Pearson χ^2^ test of association; significant at *P* < .05.

c Ever been diagnosed with arthritis (eg, rheumatoid arthritis, gout, lupus, fibromyalgia).

d Ever been diagnosed with any type of cancer.

e Ever been diagnosed with chronic heart disease, heart attack, or stroke.

f Ever been diagnosed with COPD or pulmonary disease.

g Limited in any way in any activity because of physical, mental, or emotional problems.

h Body mass index (BMI, kg/m^2^) is categorized as underweight (BMI <18.5), normal weight (BMI, 18.5–24.9), overweight (BMI, 25.0–30.0), or obese (BMI >30.0).

i Smoked more than 100 cigarettes during lifetime.

j Men having more than 14 drinks per week and women having more than 7 drinks per week.

k During the past month, other than for your regular job, did you participate in any physical activities or exercises such as running, calisthenics, golf, gardening, or walking for exercise.

l Separated, widowed, never married, a member of an unmarried couple.

m Geographic regions based on US Census (a detailed list of states included in each division is available at https://www2.census.gov/geo/pdfs/maps-data/maps/reference/us_regdiv.pdf).

Bivariate analysis of the respondents indicated that health condition, lifestyle, and demographic variables were significantly different for people reporting diabetes or prediabetes and the general population (Table 2). People diagnosed with diabetes and prediabetes were more likely to have obesity than the general public (54.0% and 47.6%, respectively, vs 29.1%), be current or past smokers (52.7% and 52.6%, respectively, vs 43.7%), have regular access to a physician (92.8% and 88.3%, respectively, vs 79.4%), and receive a regular annual checkup (86.0% and 79.2%, respectively, vs 68.7%). People with both diabetes and prediabetes reported less regular exercise (62.8% and 70.4%, respectively) compared with the general population (76.1%) (Table 2). Geographically, the distribution of diabetes and prediabetes varied. Although reporting differences existed, prevalence of prediabetes was proportionately lower in Western areas and proportionately higher in the South (Table 2).

**Table 2:** Characteristics of Respondents That Have Ever Been Diagnosed with Prediabetes (N = 63,567) or Diabetes (N = 215,441), Behavioral Risk Factor Surveillance System, 2011–2015^a^

Characteristic	General Population, %	*P* Value^b^	Prediabetes, %	*P* Value^b^	Diabetes, %	*P* Value^b^
**Survey year**						
2011	19.4	<.001	1.4	<.001	10.0	<.001
2012	20.9		3.3		10.4	
2013	19.9		3.7		10.6	
2014	20.5		4.8		10.8	
2015	19.2		—c		10.9	
**Health Condition**						
**Chronic condition**						
Arthritis^d^	26.0	<.001	43.6	<.001	49.3	<.001
Depressive disorder	17.7	<.001	27.5	<.001	26.1	<.001
Asthma	13.4	<.001	17.7	<.001	17.6	<.001
Cancer^e^	11.7	<.001	17.3	<.001	19.3	<.001
Cardiovascular disease^f^	8.7	.002	15.4	<.001	25.3	<.001
Chronic obstructive pulmonary disease (COPD)^g^	6.6	<.001	12.7	<.001	14.1	<.001
Kidney disease	2.7	<.01	3.7	<.001	8.9	<.001
Limited activity^h^	21.2	<.001	33.5	<.001	41.4	<.001
**General health**						
Excellent, Very good	52.6	.03	35.8	<.001	19.0	<.001
Good, Fair	42.7		56.4		65.5	
Poor	4.6		7.8		15.5	
**BMI^i^**						
Underweight	1.7	<.001	0.9	<.001	0.6	<.001
Normal	33.2		17.3		13.7	
Overweight	36.0		34.2		31.7	
Obese	29.1		47.6		54.0	
**Lifestyle**						
**Smoking^j^**	43.7	<.001	52.6	<.001	52.7	<.001
**Consume alcohol regularly^k^**	6.3	<.001	5.4	<.001	2.6	<.001
**Exercise regularly^l^**	76.1	<.001	70.4	<.001	62.8	<.001
**Demographics/socioeconomics**						
**Military status**						
Veteran	12.2	<.001	16.1	<.001	18.7	<.001
Nonveteran	87.8		83.9		81.3	
**Education level**						
High school graduate or less	40.2	<.001	44.4	<.001	51.5	<.001
Some college and above	59.8		55.6		48.5	
**Marital status**						
Married	55.3	<.001	57.6	<.001	56.1	<.001
Never married	23.7		15.0		11.9	
Other^m^	21.0		27.3		32.1	
**Sex**						
Male	50.5	.19	49.0	<.001	52.8	<.001
Female	49.5		51.0		47.2	
**Race/ethnicity**						
White non-Hispanic	68.1	<.001	70.5	<.001	62.6	<.001
Black non-Hispanic	11.6		15.2		16.2	
Hispanic	13.0		7.6		14.2	
Other	7.3		6.6		7.0	
**Income, $**						
<15,000	11.5	<.001	12.7	<.001	18.2	<.001
15,000 to <25,000	16.9		19.3		23.2	
25,000 to <35,000	10.8		12.1		12.8	
35,000 to <50,000	14.1		15.2		14.1	
≥50,000	46.6		40.6		31.7	
**Health care access**						
Consistent access to health provider	79.4	<.001	88.3	<.001	92.8	<.001
Annual health checkup	68.7	<.001	79.2	<.001	86.0	<.001
**Age, y**						
18–34	27.1	<.001	11.5	<.001	4.3	<.001
35–49	26.1		22.3		16.0	
50–64	28.1		38.6		40.1	
≥65	18.7		27.6		39.6	
**Geographic region^n^**						
Midwest: East North Central division	16.1	<.001	16.5	<.001	15.9	<.001
Midwest: West North Central division	6.8		5.7		6.1	
South: South Atlantic division	19.8		28.0		21.1	
South: East South Central division	5.8		12.8		7.0	
South: West South Central division	11.3		5.2		12.6	
Northeast: New England division	4.7		4.7		3.9	
Northeast: Middle Atlantic division	12.9		13.6		12.5	
West: Mountain division	7.1		6.2		6.1	
West: Pacific division	15.6		7.4		14.8	

a Values reflect weighted percentages affirmative for condition or behavior.

b *P* values based on Pearson χ^2^ test of association; significant at *P* < .05.

c Prediabetes prevalence for 2015 is not included because a large number of states did not report on prediabetes.

d Ever been diagnosed with arthritis (eg, rheumatoid arthritis, gout, lupus, fibromyalgia).

e Ever been diagnosed with any type of cancer.

f Ever been diagnosed with chronic heart disease, heart attack, or stroke.

g Ever been diagnosed with COPD or pulmonary disease.

h Limited in any way in any activity because of physical, mental, or emotional problems.

i Body mass index (BMI, kg/m^2^) is categorized as underweight (BMI <18.5), normal weight (BMI 18.5–24.9), overweight (BMI 25.0–30.0), or obese (BMI >30.0).

j Smoked more than 100 cigarettes during lifetime.

k Men having more than 14 drinks per week and women having more than 7 drinks per week.

l During the past month, other than for your regular job, did you participate in any physical activities or exercises such as running, calisthenics, golf, gardening, or walking for exercise.

m Separated, widowed, never married, a member of an unmarried couple.

n Geographic regions based on United States Census (a detailed list of states included in each division is available at https://www2.census.gov/geo/pdfs/maps-data/maps/reference/us_regdiv.pdf).

The 5-year aggregate study population was 50.5% male and 49.5% female (Table 2). People diagnosed with prediabetes or diabetes were more likely to be white non-Hispanic than the survey population (70.5% and 62.6%, respectively, vs 68.1%). Similarly, people diagnosed with prediabetes or diabetes were more likely to be black non-Hispanic than the survey population (15.2% and 16.2%, respectively, vs 11.6%) (Table 2). The risk of diabetes and prediabetes increased with age; respondents aged 18 to 34 years and 35 to 49 years had a higher proportion of prediabetes compared with diabetes (Table 2).

After adjusting for all health, lifestyle, and demographic variables, BMI and age remained most predictive in determining odds of prediabetes and diabetes. Adjusted odds of overweight among respondents with prediabetes (AOR, 1.61; 95% CI, 1.54–1.68) and diabetes (AOR, 1.77; 95% CI, 1.71–1.83) were similar. However, participants with obesity had higher adjusted odds of diabetes (AOR, 3.66; 95% CI, 3.55–3.78) than prediabetes (AOR, 2.47; 95% CI, 2.36–2.58). The most significant predictors of prediabetes, in magnitude of importance, were obesity and age, which were also predictors for diabetes, although the order of magnitude was reversed, with age followed by obesity. Multicollinearity assessment using a variance inflation factor indicated no collinearity among the variables (collinearity <4).

Adjusted odds for prediabetes showed a steady year-to-year increase from 2.4 in 2012 to 3.5 in 2014 (2015 data missing), yet diabetes prevalence remained steady for years 2011 through 2015 (Table 3). Among the 8 health conditions in this study, the unadjusted prevalence of CVD and kidney disease was higher among those with diabetes than among those with prediabetes (Table 2). After adjusting for all other variables, the adjusted odds for CVD and kidney disease remained significantly higher among those with diabetes than those with prediabetes: with CVD and diabetes, AOR of 1.56 (95% CI, 1.52–1.60), and with prediabetes, AOR of 1.06 (95% CI, 1.01–1.10); kidney disease with diabetes, AOR of 1.97 (95% CI, 1.88–2.06), and with prediabetes, AOR of 0.84 (95% CI, 0.78–0.91) (Table 3). For the other 5 chronic health conditions, the percentage prevalences and AORs for those with prediabetes and those with diabetes were comparable or slightly higher (Tables 2 and 3).

**Table 3:** Logistic Regression Model and Estimate of Maximum Likelihood for Prediabetes and Diabetes, Adjusting for Health Conditions, Lifestyle, and Demographics, Behavioral Risk Factor Surveillance System, 2011–2015

Characteristic	Prediabetes AOR (95% CI)	Diabetes AOR (95% CI)	Prediabetes Max Likelihood	Diabetes Max Likelihood
**Survey year**				
2011	1 [Reference]			
2012	2.43 (2.29–2.58)	1.03 (1.00–1.07)	4.44	0.15
2013	2.71 (2.56–2.87)	1.03 (1.00–1.07)	4.89	0.15
2014	3.48 (3.30–3.67)	1.05 (1.02–1.09)	6.18	0.25
2015	—a	1.05 (1.02–1.09)	—a	0.25
**Health Condition**				
**Chronic condition**				
Arthritis^b^	1.20 (1.16–1.24)	1.04 (1.02–1.07)	0.97	0.22
Depressive disorder	1.36 (1.31–1.41)	1.11 (1.08–1.14)	1.44	0.50
Asthma	1.14 (1.09–1.20)	1.10 (1.06–1.13)	0.56	0.38
Cancer^c^	1.10 (1.05–1.14)	0.94 (0.91–0.96)	0.36	−0.25
Cardiovascular disease^d^	1.06 (1.01–1.10)	1.56 (1.52–1.60)	0.19	1.53
Chronic obstructive pulmonary disease (COPD)^e^	1.13 (1.07–1.20)	0.92 (0.88–0.95)	0.38	−0.27
Kidney disease	0.84 (0.78–0.91)	1.97 (1.88–2.06)	−0.34	1.35
Limited activity^f^	1.08 (1.04–1.13)	1.07 (1.05–1.10)	0.40	0.35
**General health**				
Excellent, Very Good	1 [Reference]			
Good, Fair	1.38 (1.33–1.43)	2.89 (2.81–2.97)	1.94	6.44
Poor	1.18 (1.09–1.27)	4.78 (4.56–5.01)	0.42	4.04
**BMI^g^**				
Underweight	0.96 (0.82–1.13)	0.69 (0.60–0.80)	−0.06	−0.59
Normal	1 [Reference]			
Overweight	1.61 (1.54–1.68)	1.77 (1.71–1.83)	2.79	3.36
Obese	2.47 (2.36–2.58)	3.66 (3.55–3.78)	5.04	7.24
**Lifestyle**				
**Smoking^h^**	1.13 (1.10–1.17)	1.02 (1.00–1.05)	0.75	0.14
**Consume alcohol regularly^i^**	1.02 (0.96–1.10)	0.55 (0.51–0.58)	0.07	−1.82
**Exercise regularly^j^**	1.04 (1.00–1.08)	0.91 (0.88–0.93)	0.19	−0.52
**Demographics/socioeconomics**				
**Military status**				
Veteran	1.08 (1.04–1.13)	1.07 (1.04–1.11)	0.32	0.29
Nonveteran	1 [Reference]			
**Education level**				
High school graduate or less	1 [Reference]			
Some college and above	1.02 (0.99–1.06)	0.99 (0.96–1.01)	0.13	−0.09
**Marital status**				
Married	1 [Reference]			
Never married	0.96 (0.91–1.02)	1.00 (0.96–1.04)	−0.22	−0.01
Other^k^	0.98 (0.95–1.02)	0.95 (0.92–0.97)	−0.08	−0.26
**Sex**				
Male	1 [Reference]			
Female	1.01 (0.98–1.05)	0.79 (0.77–0.81)	0.08	−1.48
**Race/ethnicity**				
White non-Hispanic	1 [Reference]			
Black non-Hispanic	1.14 (1.08–1.20)	1.47 (1.42–1.52)	0.52	1.51
Hispanic	0.84 (0.78–0.92)	1.51 (1.45–1.58)	−0.70	1.71
Other	1.38 (1.29–1.48)	1.61 (1.52–1.71)	1.03	1.53
**Income, $**				
<15,000	1 [Reference]			
15,000 to <25,000	1.09 (1.02–1.16)	0.91 (0.88–0.95)	0.38	−0.44
25,000 to <35,000	1.11 (1.04–1.19)	0.83 (0.79–0.86)	0.40	−0.73
35,000 to <50,000	1.10 (1.03–1.18)	0.75 (0.71–0.78)	0.42	−1.26
≥50,000	1.01 (0.95–1.08)	0.66 (0.63–0.69)	0.09	−2.57
**Health care access**				
Consistent access to health provider	0.81 (0.76–0.86)	0.53 (0.51–0.56)	−1.06	−3.13
Annual health checkup	0.82 (0.79–0.86)	0.54 (0.53–0.56)	−1.13	−3.47
**Age, y**				
18–34	1 [Reference]			
35–49	1.61 (1.50–1.73)	2.91 (2.73–3.11)	2.56	5.77
50–64	2.23 (2.08–2.39)	6.21 (5.82–6.62)	4.42	10.08
≥65	2.28 (2.12–2.46)	9.46 (8.85–10.1)	3.95	10.75
**Geographic region^l^**				
Midwest: East North Central division	1 [Reference]			
Midwest: West North Central division	0.85 (0.79–0.90)	0.99 (0.96–1.03)	−0.51	−0.03
South: South Atlantic division	1.43 (1.36–1.51)	1.05 (1.02–1.09)	1.76	0.26
South: East South Central division	2.13 (2.01–2.26)	1.10 (1.05–1.14)	2.17	0.26
South: West South Central division	0.46 (0.42–0.50)	1.12 (1.07–1.17)	−3.02	0.44
Northeast: New England division	1.03 (0.97–1.07)	0.94 (0.91–0.98)	0.07	−0.16
Northeast: Middle Atlantic division	1.07 (1.00–1.14)	0.97 (0.93–1.01)	0.27	−0.14
West: Mountain division	0.96 (0.90–1.03)	1.01 (0.97–1.05)	−0.12	0.03
West: Pacific division	0.51 (0.48–0.54)	1.08 (1.03–1.13)	−3.01	0.33

Abbreviations: AOR, adjusted odds ratio; CI, confidence interval.

a Prediabetes prevalence for 2015 is not included because of large number of states not reporting on prediabetes.

b Ever been diagnosed with arthritis (eg, rheumatoid arthritis, gout, lupus, fibromyalgia).

c Ever been diagnosed with any type of cancer.

d Ever been diagnosed with chronic heart disease, heart attack, or stroke.

e Ever been diagnosed with COPD or pulmonary disease.

f Limited in any way in any activity because of physical, mental, or emotional problems.

g Body mass index (BMI, kg/m^2^) is categorized as underweight (BMI <18.5), normal weight (BMI 18.5–24.9), overweight (BMI 25.0–30.0), or obese (BMI >30.0).

h Smoked more than 100 cigarettes during lifetime.

i Men having more than 14 drinks per week and women having more than 7 drinks per week.

j During the past month, other than for your regular job, did you participate in any physical activities or exercises such as running, calisthenics, golf, gardening, or walking for exercise.

k Separated, widowed, never married, a member of an unmarried couple.

l Geographic regions based on United States Census (a detailed list of states included in each division is available at https://www2.census.gov/geo/pdfs/maps-data/maps/reference/us_regdiv.pdf).

The aggregate prevalence of chronic diseases for the 5-year period 2011 through 2015 was calculated in the general population and for prediabetes and diabetes (Figure). All values were significant (data not shown). The unadjusted bivariate analysis indicated that the prevalence of chronic diseases was higher among respondents with obesity who had diabetes. A higher percentage of people with prediabetes was found in the underweight (not shown), normal, and overweight categories (Figure).

**Figure:**
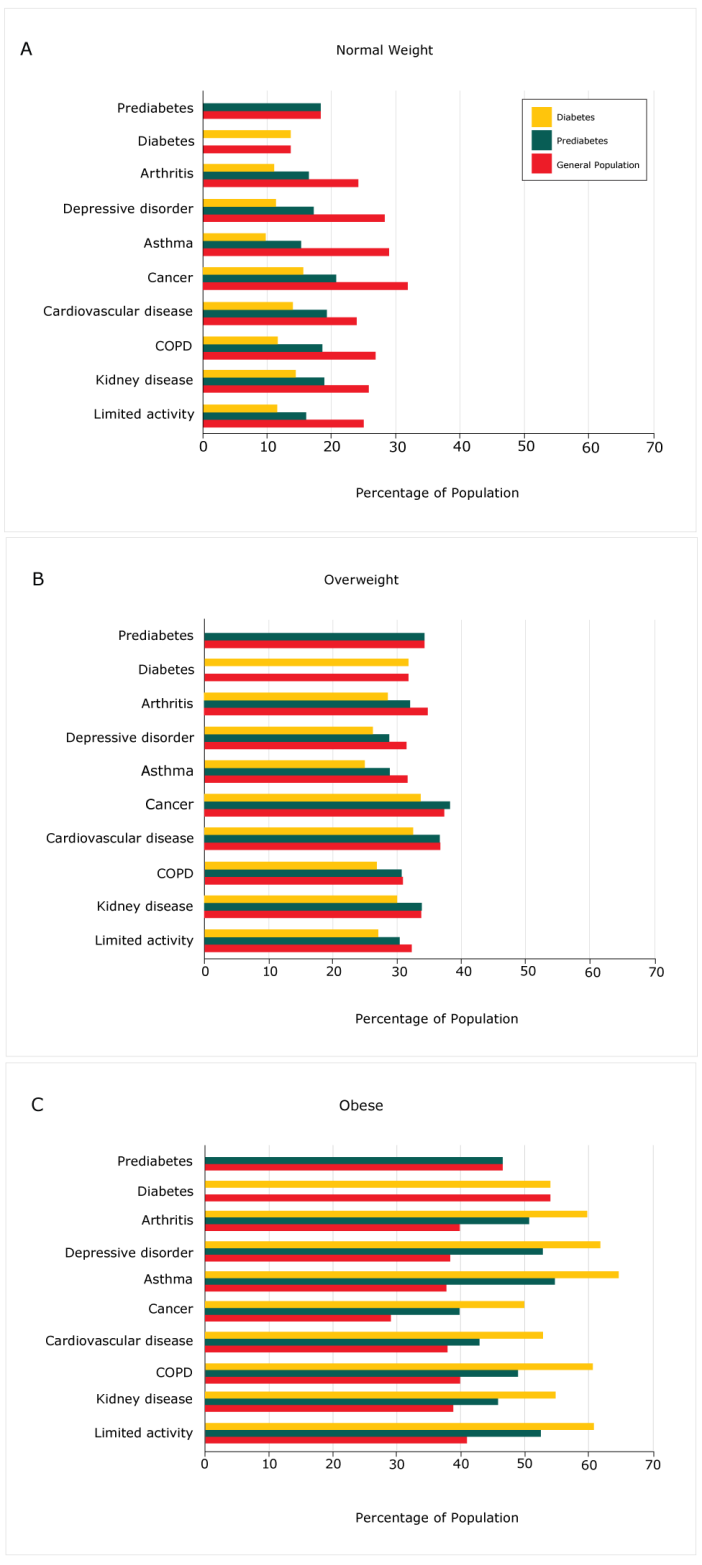
Unadjusted bivariate analysis of prevalence of chronic diseases among persons with prediabetes and diabetes by body mass index category, Behavioral Risk Factor Surveillance System, 2011–2015. Abbreviation: COPD, chronic obstructive pulmonary disease. **Table:** 

Body Mass Index Category/Chronic Disease	General Population	Prediabetes	Diabetes
**Normal weight**			
Prediabetes	18.3	18.3	0.0
Diabetes	13.7	0.0	13.7
Arthritis	24.1	16.4	11.0
Depressive disorder	28.3	17.2	11.3
Asthma	28.9	15.3	9.7
Cancer	31.9	20.7	15.7
Cardiovascular disease	23.9	19.2	14.0
COPD	26.8	18.6	11.6
Kidney disease	25.8	18.9	14.4
Limited activity	25.0	16.0	11.5
**Overweight**			
Prediabetes	34.2	34.2	0.0
Diabetes	31.7	0.0	31.7
Arthritis	34.8	32.1	28.6
Depressive disorder	31.4	28.7	26.2
Asthma	31.5	28.8	24.9
Cancer	37.4	38.3	33.7
Cardiovascular disease	36.7	36.6	32.5
COPD	30.8	30.6	26.7
Kidney disease	33.8	33.9	30.0
Limited activity	32.2	30.3	27.0
**Obese**			
Prediabetes	46.6	46.6	0.0
Diabetes	54.0	0.0	54.0
Arthritis	39.8	50.7	59.8
Depressive disorder	38.4	52.9	61.9
Asthma	37.7	54.8	64.7
Cancer	29.1	39.9	50.0
Cardiovascular disease	38.0	43.0	52.9
COPD	39.8	48.9	60.6
Kidney disease	38.9	45.9	54.9
Limited activity	40.9	52.5	60.8

## Discussion

Prediabetes is an early indicator of diabetes and contributes to the worldwide pandemic (3,7). Between 5% and 10% of people with prediabetes are estimated to progress annually to diabetes, depending on race/ethnicity and the detailed pathogenesis of their prediabetes (2,^9^,^11^). Of the estimated 86 million individuals with prediabetes in the United States, only 8% to 11.6%, or between 7 and 10 million individuals, have received a diagnosis and are aware of their prediabetes condition. Furthermore, a consistent set of chronic diseases associated with diabetes is seen in people with diagnosed prediabetes, even at lower BMI. This is alarming and may indicate a greater need for more rigorous diagnosis of prediabetes. It also raises the question of whether current treatments and interventions for prediabetes, although successful in delaying progression to diabetes, sufficiently address other chronic diseases concomitant with prediabetes. Many of the chronic health conditions included in this study are closely related to obesity, and are most prevalent among populations with obesity who have diabetes (Figure). However, the increasing frequency of these conditions among people with diagnosed prediabetes at lower BMI (normal and overweight) may signify an unwelcome trend of increased risk of comorbidities at lower BMI in prediabetes.

In an extensive meta-analysis of 16 prospective cohort studies that included more than 890,000 participants, Huang et al found that people with prediabetes at baseline had a significantly increased risk of cancer (18). Additional literature has associated increased risk for kidney disease (19), CVD (20), and arthritis (21) with prediabetes. Risk factors for diabetes and prediabetes (age, obesity, and physical inactivity) have been documented (22,23) and are confirmed in our study, with age and BMI being most highly predictive for both conditions. Conversely, regular annual checkups and access to physicians had a protective effect on diabetes. Accordingly, the focus has been on changing lifestyle habits among people with prediabetes and diabetes and using medication (24,25).

Several international trials have demonstrated the reversion from prediabetes to normoglycemia, based on lifestyle and drug-based interventions. The Finnish Diabetes Prevention Study reported average weight loss of 4.2 kg during a 3-year period using lifestyle intervention and medication (26). However, there are concerns that treating prediabetes with medication is an overtreatment of a nondisease condition and should be approached only in cases with other comorbidities, such as heart disease (27). One of the many debates about treatments of prediabetes is the question of whether the focus should be on reversing the condition or simply delaying development of diabetes. Studies suggest that prolonged duration of prediabetes can result in both microvascular and macrovascular complications of diabetes, even in the absence of overt development of diabetes (11). Our results concur with such concerns and add to the body of knowledge addressing the possible public health implications of an extended long-term prediabetes condition.

Our study has limitations. First, we used self-reported data, which were not confirmed by medical records or other health history information. Self-reported data may not reflect the continuum of disease and may better be assessed with a simple functional health assessment, which was outside the limits of this study. Furthermore, the survey questions were designed as “Have you ever…,” eliminating any distinction between prevalence and those who may have reverted to normoglycemia, resulting in possible overestimation of current prevalence. Second, self-reported diabetes does not distinguish between type 1 and type 2 diabetes; however, it is generally accepted that more than 90% of diabetes in the United States is type 2 (15). Although limitations are inherent in the depth and accuracy of any self-reported survey data, it nonetheless allows us to identify consistencies in variables common in both diabetes and prediabetes. Third, although BRFSS data encompass a large cross-section of the population, including both cellular telephone and landline telephone surveys since 2011, they still exclude or could underrepresent certain groups and races/ethnicities with language limitations, telephone access limitations, or those who are institutionalized. Fourth, reporting frequencies on prediabetes-specific questions has been inconsistent among the 50 states and the District of Columbia; some states did not report on that survey question during 1 or more of the 5 periods of this study, and in particular 39 states did not collect data on prediabetes for the 2015 survey year. As such we expect the prevalence of diagnosed prediabetes to be an underestimation and not valid for geographic region comparisons. This has also precluded us from estimating prediabetes prevalence for 2015. Another possible bias is that prediabetes overall is more prevalent than diagnosed prediabetes. As such, there may be a differential misclassification for diagnosed prediabetes with concurrent comorbidities. More prospective data may provide an excellent source to isolate the effect size of any such bias. Fifth, the nature of the cross-sectional survey prevents any extrapolation of causal relationships between the various health conditions used in this study and diabetes or prediabetes. Therefore, it cannot be determined if impaired glucose metabolism is responsible for other health conditions or perhaps caused by some combination of comorbidities included in this study. However, it is generally accepted that obesity is a common cause for most chronic health conditions. Furthermore, the self-reported diagnosis of prediabetes is likely an underestimation of actual prediabetes in the United States, because the American Diabetes Association only recommends screening for this condition starting at age 45, and then only if there are other health factors (15); similarly, adults younger than 50 may not be aware that they have diabetes. Lastly, BRFSS does not include any questions about frequency of testing the blood glucose level, glycated hemoglobin A_1c_, or any other screening or treatments of those diagnosed with prediabetes. As such, it is unknown if people with diagnosed prediabetes are getting the same or similar care as people diagnosed with diabetes.

Although much attention has been given to diagnosis of at-risk populations at the stage of prediabetes to reduce incidence of diabetes, efforts are focused on preventing prediabetes from progressing to diabetes. Implied in this attitude is the view that prediabetes has lower rates of morbidity compared with diabetes. However, the validity of this assumption is not clear. This study highlights that many chronic disease conditions are present at high rates in prediabetes and that a prolonged period of prediabetes does not necessarily reduce the risk of certain comorbidities compared with diabetes. Our results suggest that there may even be an increased risk at lower BMI among people with prediabetes to present with other chronic comorbid health conditions. In light of potential comorbidities that may occur in this at-risk population, substantial effort should be considered to identify prediabetes at a lower BMI and younger age, where rigorous attempts to reverse prediabetes to normoglycemia could prove far more beneficial in promoting public health.
